# Detection and Quantification of Adulteration in Krill Oil with Raman and Infrared Spectroscopic Methods

**DOI:** 10.3390/molecules28093695

**Published:** 2023-04-25

**Authors:** Fatema Ahmmed, Keith C. Gordon, Daniel P. Killeen, Sara J. Fraser-Miller

**Affiliations:** 1 Te Whai Ao-Dodd-Walls Centre for Photonic and Quantum Technologies, Department of Chemistry, University of Otago, P.O. Box 56, Dunedin 9016, New Zealand; fahmmed@massey.ac.nz; 2 The New Zealand Institute for Plant and Food Research Limited, P.O. Box 5114, Port Nelson, Nelson 7043, New Zealand; daniel.killeen@plantandfood.co.nz

**Keywords:** adulteration, chemometrics, PCA, SVM, PLSR, omega-3 fatty acids, marine lipid, vibrational spectroscopy, Raman spectroscopy, infrared spectroscopy, low level data fusion

## Abstract

Raman and infrared spectroscopy, used as individual and low-level fused datasets, were evaluated to identify and quantify the presence of adulterants (palm oil, PO; ω-3 concentrates in ethyl ester, O3C and fish oil, FO) in krill oil. These datasets were qualitatively analysed with principal component analysis (PCA) and classified as adulterated or unadulterated using support vector machines (SVM). Using partial least squares regression (PLSR), it was possible to identify and quantify the adulterant present in the KO mixture. Raman spectroscopy performed better (r^2^ = 0.98; RMSEP = 2.3%) than IR spectroscopy (r^2^ = 0.91; RMSEP = 4.2%) for quantification of O3C in KO. A data fusion approach further improved the analysis with model performance for quantification of PO (r^2^ = 0.98; RMSEP = 2.7%) and FO (r^2^ = 0.76; RMSEP = 9.1%). This study demonstrates the potential use of Raman and IR spectroscopy to quantify adulterants present in KO.

## 1. Introduction

The presence of adulterants in food stuffs can be a serious food safety issue [1]. Krill oil is a marine-derived bioactive lipid that is extracted from oceanic krill (*Euphausia superba*) and commercially available in the market globally. Krill oil contains considerable amounts of long chain ω-3 polyunsaturated fatty acids, in particular eicosapentaenoic acid (EPA), docosapentaenoic acid (DPA) and docosahexaenoic acid (DHA) [2], which have numerous health benefits [3,4] and are considered as functional oils to fortify food [5], pharmaceutical [6,7] and cosmetics products [8]. Importantly, in KO, many of these fatty ω-3 acids are esterified to phospholipids, unlike standard fish oils or oils derived from terrestrial plants [9,10,11]. The phospholipid form of ω-3 fatty acids (PL-ω3) is reported to have higher bioavailability compared to the triglyceride or ethyl ester forms of ω-3 fatty acids for delivery to the heart, liver, kidney and brain [12,13]. Efficient DHA uptake by the brain has only been demonstrated with PL-ω3 sources such as krill oil and is comprehensively reviewed elsewhere [14]. The extraction of phospholipid from krill oil is a multistep process that requires a large amount of oceanic krill. For example, 100 g of krill is required to obtain 9.26 to 19.61 g of oil depending on the extraction methods used [15]. Krill oil is produced from a limited set of krill species (mostly *E. superba*), whereas fish oil can be produced from a wide variety of fish species, either whole— e.g., Peruvian anchoveta (*Engraulis ringens*)—or from processing by-products. Krill oil products tend to be more expensive than fish oil because of the perceived higher health benefits [16].

Adulteration is a common fraudulent activity in the marine oil industry, which has been reported previously [17,18]. One such example is the adulteration of cod liver oil with low-quality shark oil [19]. On multiple occasions, the FDA (Food and Drug Administration) has identified generic fish oil adulteration in both cod liver and halibut liver oils [20]. Fish oil supplements might contain a considerable amount of ethyl ester and do not meet the label claimed content of ω-3 PUFA [21]. The quantity of valuable bioactive compounds (EPA, DHA) is reduced in the adulterated product. Therefore, a rapid and non-destructive method of checking krill oil for adulteration is desirable.

Vibrational spectroscopic methods, such as infrared (IR) and Raman spectroscopy, are a candidate technology for non-destructive authentication of krill oil capsules. These techniques have been demonstrated previously as a method to quantify bioactive compounds in marine oils. Killeen et. al. quantified total PUFA content and detected ethyl ester in intact fish oil capsules using Raman spectroscopy [22]. Both IR and Raman spectroscopic methods have been demonstrated to classify the lipid class (TAG, EE) in fish oil with 100% accuracy [23].

IR spectroscopy has been demonstrated previously to detect and quantify fish oil adulteration. The adulteration of cod liver oil (CLO) with vegetable oils (1–50% of: corn oil (CrO); canola oil (CnO); walnut oil (WO) and soybean oil (SO)) was evaluated using IR spectroscopy with multivariate methods for quantification (PLS and PCR) and discrimination analysis (DA) [24]. The PLS models for the independent test set also gave lower prediction errors (RMSEP = 1.35–1.75%) than PCR models (RMSEP = 1.28–1.87%), indicating the suitability of the model for quantifying the level of adulterant (vegetable oils) in cod liver oil. DA analysis could successfully classify pure CLO and adulterated CLO samples using nine (9) principal components [24]. IR spectroscopy has also been reported for the detection and quantification of palm oil (PO) adulteration mixed at different concentrations, 0–100% *v*/*v*, with Patin fish oil (PFO) [25]. IR spectroscopy with quantitative analysis methods (PLS, PCR) was implemented to quantify the level of PO adulteration, and the result found high r^2^ (0.99) and low prediction error values (RMSEC: 0.77–5.50%, 0.88–2.54%; RMSEP: 2.43–3.59%, 2.01–2.61%, respectively). Discriminant analysis also successfully classified pure PFO from the adulterated PFO with 100% accuracy [25].

These studies demonstrate the potential use of Raman and IR spectroscopy for the detection of various adulterants in marine oils. The use of Raman and IR spectroscopy has also been demonstrated for the detection of adulterants in edible oils (i.e., olive oil, coconut oil, sunflower oil) [26,27,28].Very few vibrational spectroscopic studies have focused on fish oil [25,29]; one of these is our recent study [30], where we investigated Raman and IR separately and as a fused dataset to quantify adulterants (PO, O3C and FO) in fish oil samples (cod liver oil and salmon oil). Our research concluded that the SVM classification analysis successfully classified the adulterants and the PLSR model quantified the amount of adulterant in the fish oil samples. However, to date, the feasibility of using vibrational spectroscopy and multivariate methods techniques for detecting adulteration of krill oil has not been reported.

In the present study, two complementary vibrational spectroscopic methods (Raman and IR spectroscopy) and low-level fusion of these datasets was used to:

(1) Identify the presence of adulterants (palm oil (PO); ω-3 concentrates in ethyl ester (O3C) and fish oil (FO)) in krill oil,
(2) Quantify the amount of adulterant present and
(3) Identify the most promising spectroscopic technique or combination thereof for the quantification of adulterant(s) in krill oil.

## 2. Results and Discussion

### 2.1. Raman and Infrared Spectral Features of the Oil Samples

**Figure 1:**
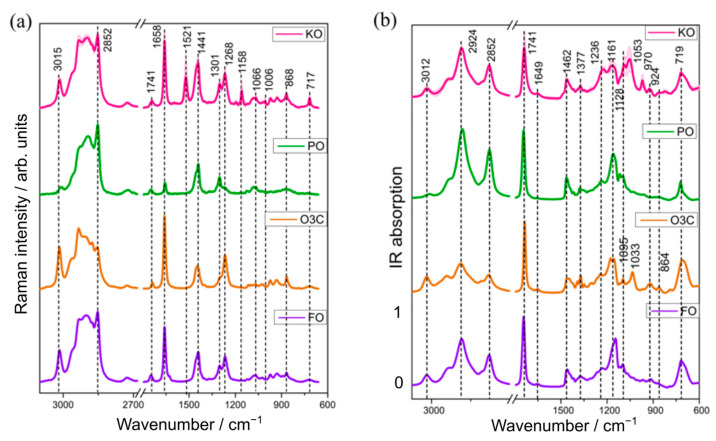
The mean (±St. Dev) Raman (**a**) and infrared (**b**) spectra of krill oil (n = 6 samples) and the three adulterants studied (palm oil, ω-3 concentrates in ethyl ester, fish oil (FO)).

The mean (±standard deviation) Raman and IR spectra obtained from the different varieties of KO and adulterant samples (PO; O3C and FO) are presented in Figure 1. Visually, the Raman spectra of PO, O3C and FO contain the same spectral bands relating to triacylglycerols, but ratios of these bands vary depending on the degree of saturation/unsaturation of constituent fatty acids, as reported previously [31,32]. On the other hand, krill oil is visually distinct from these oils with the additional bands at 1521 (conjugated C=C stretching), 1158 (C-C stretching) and 1006 (Me rocking) cm^−1^, which are attributed to astaxanthin [33]. The Raman spectra of KO, O3C and FO showed relatively intense bands at 3015 (-C=C-H stretching), 1658 (C=C stretching) and 1268 cm^−1^ (=C-H deformation) [34], characteristic of higher concentrations of unsaturated fatty acids compared to PO. This is consistent with the higher concentrations of unsaturated fatty acids in marine oils, relative to (most) plant-derived oils [35].

A low degree of unsaturated fatty acids in palm oil also explains the absence of characteristic lipid bands at 3015 cm^−1^ (-C=C-H stretching), 1268 cm^−1^ (=C-H deformation) and around 868 cm^−1^ (C-C stretching) in PO (Figure 1a). Raman spectral signatures of KO, PO and FO showed relatively higher peak intensities at 2852 (-CH (CH2) stretching), 1441 (scissoring vibration of -CH2) and 1301 cm^−1^ (bending modes of -CH2) than O3C, indicating a higher concentration of saturated fatty acids in the naturally occurring oils compared with the ω-3 concentrates in the ethyl ester format.

The IR spectra from the KO, PO, O3C and FO samples (Figure 1b) all contained the characteristic bands for fatty acids (FA) at 3012 (-HC=CH- stretching of cis-alkene), 2924 (-CH2- stretching of methylene), 2852 cm^−1^ (-CH2- stretching of methylene) and other lipid signals at 1741, 1462, 1237 and 1095 cm^−1^ (C-O stretching vibration). Rohman and Che Man reported and assigned lipid bands in various oil samples (cod liver oil, canola oil, soybean oil, walnut oil) that are in agreement with this study [36]. The infrared absorption of KO, O3C and FO samples had higher peak intensities at 3012 cm^−1^ than PO, indicating a higher concentration of unsaturated FAs. As mentioned above, marine-derived lipids are reported to contain higher levels of long chain ω-3 fatty acids than terrestrial-derived lipids [14], which is consistent with the more intense peak for the KO, O3C and FO samples than the plant-derived oil, PO. The band intensities at 2924 and 2852 cm^−1^ were found to indicate a higher concentration of saturated FAs in PO compared to the other three oil samples (KO, O3C, FO), which is consistent with the Raman spectral features (Figure 1a). This observation is in good agreement with existing literature [37], which reports high levels of saturated fatty acids (43–50% of the total fatty acids) in PO. The spectra of KO, O3C and FO had very weak bands around 1649 cm^−1^ (C=C stretching of cis-olefins), 924 cm^−1^ (=C-H deformation of trans olefins) and around 700 (cis=C-H symmetric rocking) cm^−1^, which are mostly absent in PO [24,25]. Other bands at 1462, 1236 and 1377 cm^−1^ correspond to CH2 and CH3 bending, respectively, while signals at 1161 and near 1095 cm^−1^ were assigned as C-O stretching of esters, which has also been observed previously in krill oil and fish oil samples [38,39]. Thus, the spectral variation between KO and the three adulterants observed in these spectral regions was further analysed to develop automated detection and quantification models of selected oils (PO, O3C, FO) as adulterants in KO samples.

### 2.2. Principal Component Analysis (PCA)

In this study, PCA was used as an exploratory tool to visualize the variation in the data matrix obtained by the Raman and IR spectroscopic datasets.

**Figure 2:**
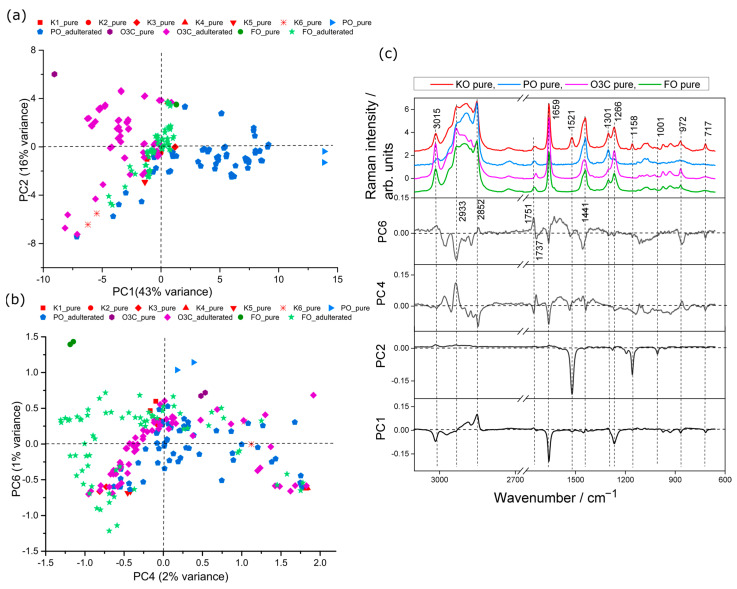
PCA scores plots of krill oil samples adulterated with PO, O3C and FO samples measured using Raman. (**a**) PC1 (43% explained variance) versus PC2 (36% of the spectral variance), (**b**) PC4 (2% explained variance) versus PC6 (1% explained variance) and (**c**) associated loading plots.

PCA of the Raman spectral data from six krill oil samples, three adulterants (PO, O3C, FO) and all the adulterated mixtures was carried out. The scores plots for this analysis are presented in Figure 2. The first two PCs explained 82% of the total variance between samples (PC1, 43%; PC2, 36%; PC4, 2% and PC6, 1%). The PCA scores plot separated adulterants from pure marine oil samples (Figure 2a). From the scores plot (PC1 versus PC2) in Figure 2a, it is observed that PO and PO adulterated samples clustered in positive PC1 space, whereas O3C and O3C adulterated samples trended to negative PC2 space. FO adulterated samples tended to cluster in neutral PC1 space amongst the KO samples. Pure KO samples tended to cluster in neutral and negative PC1 space. PO and O3C were well-separated in PC1 space, indicating higher relative spectral variance of PO and O3C in KO than FO. The loadings plot presented in Figure 2c provided insight into the spectral variances contributing to this separation. The major bands in negative PC1 space (Figure 2c) are associated with unsaturated FAs (3015, 1659 and 1266 cm^−1^, vinyl group) and other lipid signals at 972 (C=C, bending), 866 (C-C, stretching) and 717 cm^−1^ (-CH2, rocking mode of olefins), whereas the positive PC1 loaded against saturated FAs at 2852 cm^−1^. This is consistent with the ω-3 polyunsaturated fatty acid concentrations, which were highest in O3C and lowest in PO.

The second PC is separating all pure adulterants (positive PC2 space) from krill oil samples (negative and neutral PC2 space), shown in Figure 2a. Inspection of the associated loadings (Figure 2c) shows strong negative loading features (PC2) at 1521, 1158 and 1006 cm^−1^ characteristic of astaxanthin, whereas no strong positive loading features were observed. These loadings are associated with relative levels of astaxanthin content, with more negative PC2 scores associated with higher astaxanthin signals.

The FO and FO adulterated samples do not show signs of separation from the krill oil samples till much later PCs (PC4 vs. PC6, Figure 2b); they dispersed across the PC4 space but tended to clustered in negative PC4 space and towards positive PC6 space (Figure 2b), whereas the other sources of variance in these PCs appeared to be associated with differences between KO batches with the other adulterants and adulterated samples sitting neutral to these KO samples. PCs 4 and 6 explained minor levels of spectral variation (3% in total), which demonstrated the minor contribution towards spectral variation associated with FO signals compared to the other oils in the dataset. The negative PC4 spectral features (Figure 2c) are assigned as lipid (2852, 1659, 1441 and 717 cm^−1^) and weak astaxanthin (1521, 1158, 1001 cm^−1^) signals, whereas positive PC4 (Figure 2b) are correlated with few lipid signatures (2933, 972 cm^−1^) and an ester carbonyl stretch (1737 cm^−1^). Similar to the PC4 loading plot, the positive PC6 loading corresponded to unsaturated (3015 cm^−1^) lipid signals, saturated (2852 cm^−1^) lipid signals and an ester carbonyl stretch (1751 cm^−1^), whereas negative PC6 loading attributed to lipid and astaxanthin features.

PC1 was dominated by the difference in signals from PO and O3C, and, therefore, these results appear satisfactory for building models to detect adulteration from these two adulterants. The first two PCs did not directly describe variance between the adulteration of KO with FO, but minor spectral variance associated with PC4 (2%) and PC6 (1%) showed some detectable differences. The FO will be the most challenging to detect and quantify of the adulterants studied here but still worthwhile exploring.

**Figure 3:**
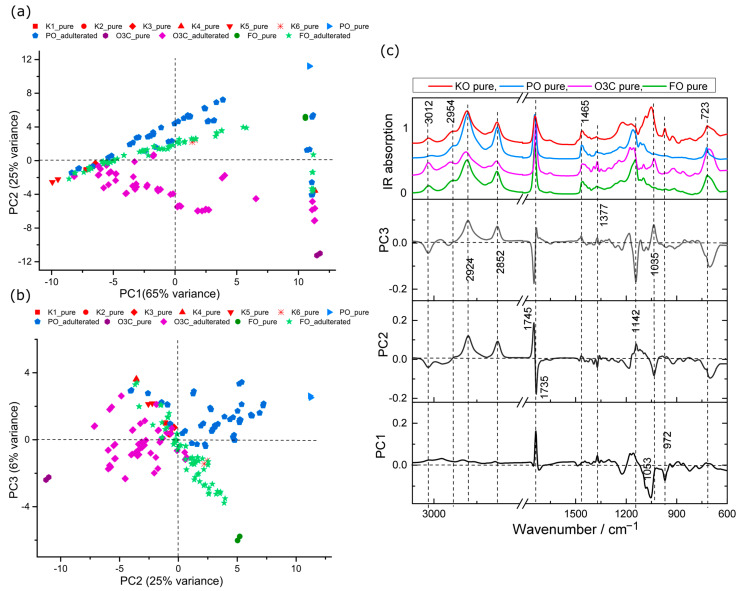
PCA scores plots of krill oil samples adulterated with PO, O3C and FO samples measured using IR. (**a**) PC1 (65% explained variance) versus PC2 (25% explained variance), (**b**) PC2 (25% explained variance) versus PC3 (6% explained variance) and (**c**) associated loading plots.

PCA analysis of IR spectral data obtained from pure oils and the adulterated samples were also carried out. The scores plots are shown in Figure 3 and the associated loadings in Figure 3c. The first three PCs accounted for 96% of the total variance among samples (PC1, 65%; PC2, 25% PC3, 6%). The scores plot separated pure and adulterated samples as in Figure 3a,b, in agreement with Raman analysis (Figure 2).

The scores plot of PC1 versus PC2, shown in Figure 3a, revealed that the spectra collected from FO and FO adulterated samples tended to cluster in neutral PC2 space. The O3C and O3C adulterated samples tended to cluster in negative PC2 space, whereas PO and PO adulterated samples tended towards the positive portion of PC2 space from the location of the pure KO that was adulterated. IR features of PO and O3C adulterants were also associated with higher spectral variance, indicating a big spectral difference of PO and O3C adulteration in KO compared to FO adulteration.

The associated loadings plots presented in Figure 3c provide insight into the spectral variances contributing to this separation. The main spectral contribution in the positive PC1 features were observed at 1735 cm^−1^ (C=O stretching, ethyl ester) and 1166 cm^−1^ (C-O stretching) [23,40], whereas the most spectral influence in the negative PC1 features were related to symmetric phosphate diester stretching in PO2 (1095 cm^−1^), asymmetric ester stretching in C-O-P (1060 cm^−1^) and various HC=CH vibration (980–950 cm^−1^) [40,41,42], indicating phospholipid signals. The second PC separated mostly the O3C adulterant samples, depending on characteristic ester shifting (1745 cm^−1^) and CH2 bending (1142 cm^−1^). O3C contains lower saturated fatty acids than PO [23], which explains the reason for the spectral variance for saturated FAs at positive PC2 loadings (2924, 2852 cm^−1^) from PO samples (Figure 3c). The negative PC2 loading exhibits spectral features consistent with lipid signals (3012, 1735, 1377, 1035 and 723 cm^−1^) (Figure 3c) mostly derived from O3C adulterants [43].

The score plot, shown in Figure 3b, revealed that the pure and PO adulterated KO samples tended to group in positive PC3 space, whereas FO trended towards negative PC3 space with increasing FO adulteration.

For the loadings of the third PC, the major positive spectral features at 2924, 2852 and 1035 cm^−1^ (Figure 3c) were associated with saturated C-H stretching and ester C-O stretching, whereas inversely loaded spectral features were indicative of lipid (3012, 1142 cm^−1^), ester (1745 cm^−1^) and methylene groups (723 cm^−1^), respectively [44].

The differences associated with PO and O3C adulteration are detected in the first two PCs, indicating these oils have distinct and readily detected features in comparison to the KO. This was also observed in the Raman data. Based on these PCA, the FO may be more readily detected with IR than Raman, as spectral variance associated with FO from the other oils is detected in an earlier PC (indicating a higher portion of detected spectral variance) with IR when compared to the Raman data.

Importantly, no astaxanthin bands were detected in ATR-IR spectra, and no separation between samples based on astaxanthin content occurred in PCA scores plots. This is because Raman spectroscopy is extremely sensitive to carotenoids like astaxanthin, giving rise to intense vibrational bands from these compounds despite its extremely low concentration [38].

### 2.3. SVM Classification of Raman and IR Data

Based on the trends observed in the PCA scores space, it was deemed that this was a promising dataset to explore the identification (classification) and quantification of adulterants in KO. SVM classification was conducted for both the Raman and IR datasets, alone and in combination, to identify the presence and type of adulterants found in the samples. The use of this classification step will act as a filter to ensure that the samples then can have the appropriate quantitative model applied for the adulterant present. The decision tree/workflow for evaluating unknown samples is presented in Figure S2.

SVM classification models were developed for Raman and IR spectra as individual and fused datasets. All three SVM models gave 100% calibration accuracy and between 86 and 96% cross-validation accuracy for the training dataset. The independent test set was then predicted using the developed SVM models to provide a more realistic measure of the likely performance on unknown samples.

The SVM classification model using the Raman data gave a classification accuracy of 76% for the independent test dataset. Inspection of the associated confusion matrix (Table S3) and sensitivity and specificity to individual classes (Table S4) showed that the misclassifications tended to occur with KO classifying as PO, some O3C adulterated samples classifying as adulterated with FO and some FO adulterated samples classifying as adulterated with PO.

The SVM model of the IR data yielded a test set accuracy of 79% with misclassifications of samples with low concentrations (<15%). The fused (Raman plus IR) SVM model provided the most promising results with a test set accuracy of 84%. The misclassifications in these data were associated mostly with random concentration, which might be also be attributed to the presence of different levels of astaxanthin content in various krill oil samples, which was recently reported to vary in commercial krill oils from 212–693 micrograms per gram of oil [2].

### 2.4. Quantitative Measurements of Krill Oil Adulteration

Partial least square regression (PLSR) analysis was performed to quantify the adulterated oil content (PO, O3C, FO) in krill oil samples. Individual PLSR models were developed for each adulterant to assess the feasibility of Raman and IR spectroscopy, used individually and fused, to quantify adulterant levels in krill oil.

These PLSR models were then evaluated with the test set data in two ways.

(1) Running the samples through the appropriate model based on prior knowledge of the adulterant used. This has the limitation that it does not reflect how a truly unknown sample will perform if we have no prior knowledge of the adulterant present.
(2) Run the test set data through the SVM classification model to evaluate the adulterant present, then feed those data through the appropriate PLSR quantitative model (see Figure S2 for the workflow). This has the advantage of no user input in the selection of the PLSR model used and will reflect a likely workflow in a truly unknown sample.

The second test set scenario (pre-evaluated with SVM classification) is what is discussed in detail below; however, the results of the first scenario are also recorded for completeness in Table 1.

#### 2.4.1. Spectroscopic Estimation of % PO in Krill Oil

**Figure 4:**
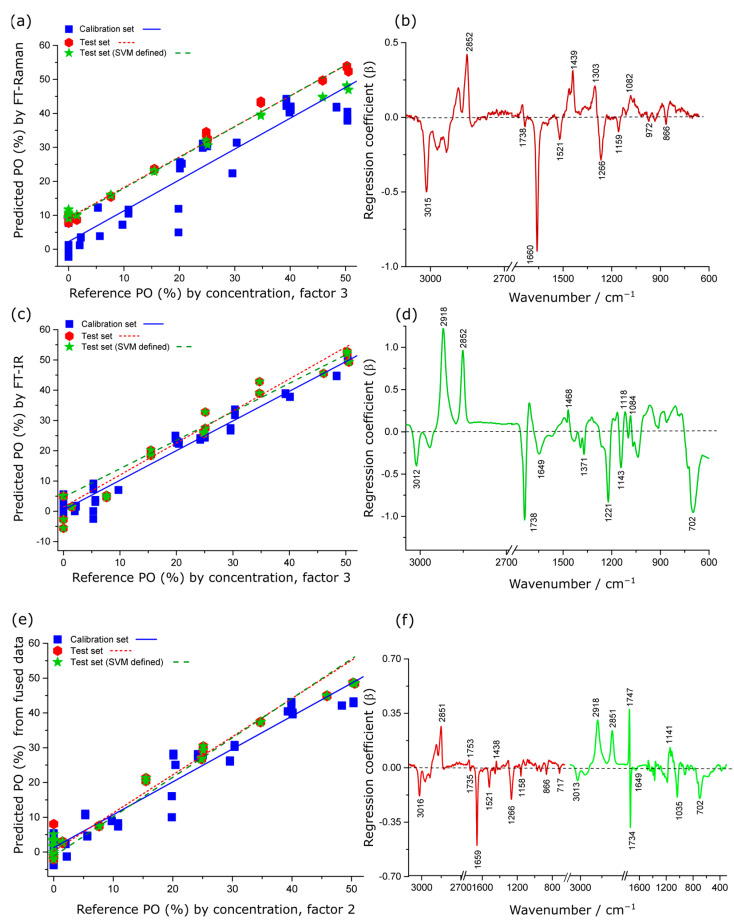
PLSR actual versus predicted concentration scatter plots and regression coefficients for quantitative prediction of PO concentration in krill oil by Raman (**a**,**b**), IR (**c**,**d**) and low-level fused Raman plus IR data (**e**,**f**). In (**c**–**e**), the red colour indicates the coefficients originating from the Raman spectra, whereas the green colour indicates coefficients originating from the IR spectra.

The PLSR models developed from the IR spectral data for quantifying PO concentration in KO performed better (RMSEP = 3.5%, r^2^ = 0.96) than the model obtained from Raman spectral data (RMSEP = 7.7%, r^2^ = 0.85) in terms of both prediction error and linearity (Table 1). In the PO model for the Raman data, the regression coefficient explained 91% of the total variance in the dataset with the first three factors (Figure 4b). The negative regression coefficient features were observed at 3015, 1660 and 1266 cm^−1^ (vinyl groups of lipid bands) and features at 1521 and 1159 cm^−1^ (astaxanthin signatures), which are consistent with the spectroscopic features from krill oil. The positive coefficients were mostly associated with characteristic lipid (2852, 1439, 1303 cm^−1^) and carbonyl signatures (1738 cm^−1^, ester bond; 1082 cm^−1^, triglycerides and cholesterol), derived from PO, as shown in Figure 4b. These loadings were in excellent agreement with the vibrational assignments of the pure PO sample, as discussed above.

For the PLSR of the IR data, the regression coefficient explained 97% of the total variance in the dataset with the first three factors (Figure 4c). The positive regression coefficient features are associated with PO contribution whereas the negative features are consistent with KO features.

The PLSR models produced from low-level fused data showed significant improvement (RMSEP = 2.7%, r^2^ = 0.98) compared to the individual Raman technique (RMSEP = 7.67%, r^2^ = 0.85) and the IR technique (RMSEP = 3.5%, r^2^ = 0.96). The interpretable regression coefficient was consistent with the same bands in the individual Raman and IR regression coefficient plots but with subtle changes in the peak intensities as spectroscopic quantification of % PO in krill oil.

It is possible to derive *LOD* and *LOQ* form these data. There are a number of methods of determining these values for data that is multivariate in nature [45,46]. We have used the equations [47,48]:

$$LOD=\frac{3.3\sigma }{S}\;LOQ=\frac{10\sigma }{S}$$

$$LOQ=\frac{10\sigma }{S}\;LOQ=\frac{10\sigma }{S}$$

where σ is the standard deviation of the regression fit of the test set with SVM (that is pre-screened for adulterant) and *S* is the slope of the validation curve. For this adulterant the % values are 9 and 28 for *LOD* and *LOQ* respectively for the fused data—which gave the best performance.

#### 2.4.2. Spectroscopic Estimation of % O3C in Krill Oil

**Figure 5:**
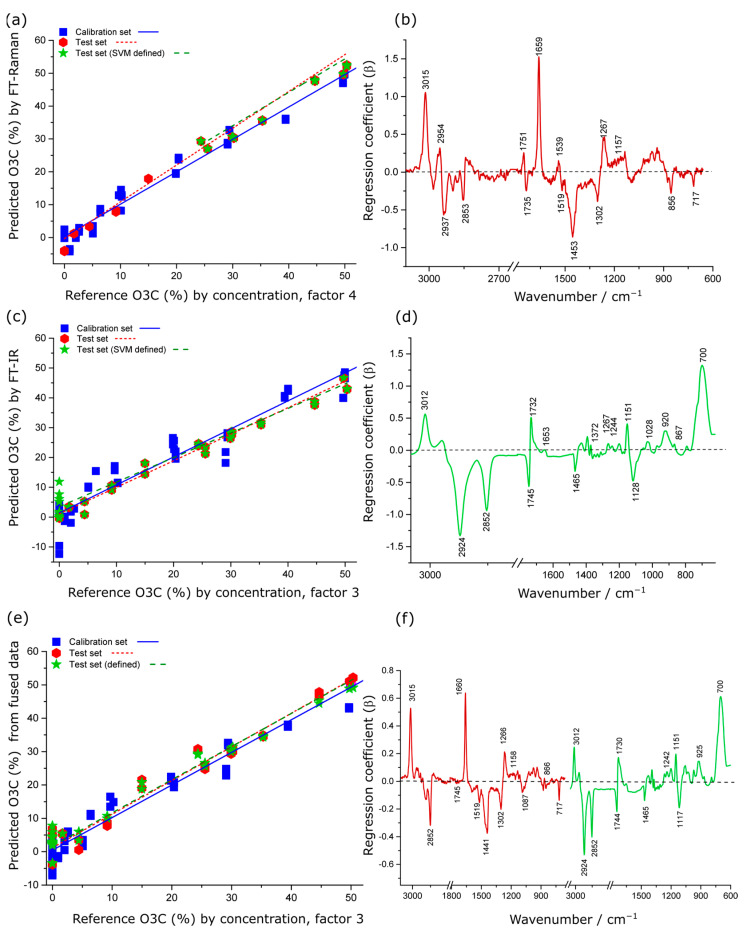
PLSR actual versus predicted concentration scatter plots and regression coefficients for quantitative prediction of O3C concentration in krill oil by Raman (**a**,**b**), IR (**c**,**d**) and low-level fused Raman plus IR data (**e**,**f**). In (**c**–**e**), the red colour indicates the coefficients originating from the Raman spectra, whereas the green colour indicates coefficients originating from the IR spectra.

The models developed from Raman spectral data for predicting the O3C concentration showed strong positive correlation with the reference values with minimum prediction error (RMSEP = 2.3%, r^2^ = 0.95), whereas the IR spectral data provided similar coefficient of determination (r^2^ = 0.95) but with higher root mean squared error of prediction (RMSEP = 4.2%) (Table 1). In the % O3C model for Raman and IR data, the regression coefficient explained 98% (Figure 5b) and 93% (Figure 5d) of the total variance in the dataset by the first four/two factors, respectively.

In regression coefficient plots for the Raman data (Figure 5b), the positive spectral variance at 3015, 1659 and 1267 cm^−1^ corresponded to the higher proportion of unsaturated FAs in O3C compared to KO, whereas the negative spectral variance at 1519, 1302 and 717 cm^−1^ was almost entirely associated with KO spectral features. The characteristic carbonyl shift at around 1735 cm^−1^ is consistent with alkyl acylation, reported previously for O3C [22,23,49].

In addition, the PLSR model produced from low-level fused data gave intermediate model performance for quantification of O3C concentration in krill oil when compared to the models developed from the individual spectroscopic techniques (Raman and IR), as presented in Table 1. In the O3C model for low-level fused data, the regression coefficient explained 93% of the total variance in the dataset by the first three factors (Figure 5f). The coefficients displayed similar band assignments for the individual spectroscopic technique and fused data model. However, the peak intensity of the spectral features varied in the fused model, likely reflecting slight differences in the weightings of band contributions when both techniques are reflected in the PLSR model. Therefore, the data fusion approach did not enhance the model performance in this case.

For the best-performing model (using the Raman data, Table 1) the *LOQ* and *LOD* are 8 and 24% respectively.

#### 2.4.3. Spectroscopic Estimation of % FO in Krill Oil

For the individual methods, the IR PLSR model for predicting FO concentration in krill oil displayed a better performance (RMSEP = 9.6% and r^2^ = 0.73) than the Raman-based model (RMSEP = 15.6% and r^2^ = 0.31). The compositional similarity of FO to krill oil is the likely reason for reduced accuracy when quantifying FO concentration in KO. These results also indicated that the test set is offset from the model set, which could be attributed to the difference in astaxanthin levels between KO batches. As there are minimal compositional differences in these samples (besides astaxanthin content), the differences in astaxanthin levels contribute to the prediction of FO content, making the predictions sensitive to astaxanthin levels in the initial KO sample. The PLSR model performance for the quantification of FO in KO are presented in Table 1.

In the FO PLSR models for the individual Raman and IR data, the regression coefficients explained 96% of the total variance in the dataset by the first four and three factors, respectively (Figure S3b,d). The regression coefficients plot for the Raman spectral PLSR model showed that the positively loaded spectral features at 3014, 1751, 1661, 1515, 1158 and 1266 cm^−1^ and the negatively loaded spectral features at 2852, 1733, 1455 and 717 cm^−1^ were found as main contributors to the prediction plot (Figure S3b). These characteristic bands were mainly attributed to different vibrational modes of lipid composition in the oils. The spectral shifts at 1515 and 1158 cm^−1^ were assigned as astaxanthin content in krill oil.

The IR regression coefficient plot revealed that the negatively loaded spectral bands at 2924, 2854, 1734, 1465, 1372 and 1221 cm^−1^ and positively loaded features at 3015 and 1140 cm^−1^ have a strong influence on the prediction plot (Figure S3d).

When the spectral datasets were fused, the PLSR model for FO concentration gave a modest improvement in prediction performance (RMSEP = 9.1% and r^2^ = 0.76) compared to Raman and IR used alone. For the best-performing model (the fused data, Table 1), the *LOQ* and *LOD* are 34 and 100%, respectively.

In the FO model for low-level fused data, the regression coefficient explained 86% of the total variance in the dataset by the first two factors (Figure S3f), which is consistent with the band assignment for the individual spectroscopic technique.

These results demonstrated that the PLSR models obtained from spectroscopic (Raman, IR) analysis are able to quantify the percentage of PO, O3C and FO as oil adulterants in krill oil samples with varying levels of associated error. The data fusion approach has the potential to enhance model performance significantly by integrating the assessed information from individual techniques into a single model matrix and is demonstrated to be advantageous for PO and FO quantification. However, O3C was better quantified with Raman spectroscopy used alone.

## 3. Materials and Methods

The sample preparation has been reported previously [30]. Briefly, six different brands of krill oil (KO) and three different adulterants (palm oil (PO), ω-3 concentrates in ethyl ester (O3C) format and fish oil (FO, triacylglycerols, 18:12 EPA: DHA)) were purchased from local and online pharmacies available in New Zealand. A summary of the oils, name of product, company, place of manufacture, batch and use by date are presented in the supplementary information (Table S1 and Figure S1). A calibration sample set (n = 54) was prepared using four batches of krill oil (K1, K2, K4 and K6) and combining these with a range of concentrations of adulterants (PO, O3C and FO), with concentrations ranging from 0–50.0% (*w*/*w*). An independent test set (n = 38) was derived from the two remaining krill oil batches (K3, K5) adulterated in the same manner to validate the calibration set. All samples were mixed by vortexing, then stored in 4 mL glass vials at −20 °C until spectral measurement collection to avoid further oxidation or structural changes. The details of the compositional mixtures used in the model and independent test sets are given in Table S2.

### 3.1. Spectroscopic Methods

Raman spectra were collected using a MultiRAM Fourier transform (FT) Raman spectrometer (Bruker Optics, Ettlingen, Germany) equipped with a liquid nitrogen-cooled Ge detector (D418T) and a Nd:YAG continuous wave laser emitting at 1064 nm. The spectral collection was controlled using OPUS software V.7.5 (Bruker Optics, Ettlingen, Germany) over the 4000–200 cm^−1^ spectral window. Sample measurements were performed inside the 4 mL glass vials with a 180-degree backscattering arrangement at 300 mW laser power with a defocused aperture (1–2 mm ⌀ spot size), 128 co-added scans and spectral resolution of 4 cm^−1^. These parameters were selected based on the methods reported in our previous work [22,30,38,50].

Infrared spectra were collected using a Vertex 70 FT-IR spectrometer (Bruker Optics, Ettlingen, Germany) equipped with a diamond attenuated total reflectance (ATR) sampling accessory (GladiATR, Pike Technologies, Madison, WI, USA). Samples (approximately 10 μL) were placed on the ATR crystal and spectral information from the spectral window 4000−300 cm^−1^ were acquired with OPUS software V.7.5 (Bruker Optics, Ettlingen, Germany). Background spectra were acquired from the cleaned blank ATR crystal prior to replicate sample acquisition. Each sample was measured in duplicate and spectral acquisitions were the average of 32 scans with a spectral resolution of 4 cm^−1^. These parameters were selected based on the methods reported elsewhere [30,38,50]. No evidence of sample degradation was detected during the experiments.

### 3.2. Spectral Pre-Processing and Data Analysis

Each spectroscopic technique required slightly different pre-processing over different spectral regions due to the different vibrational modes detected, experimental artefacts, noise, spectral intensity and baseline effects.

Raman spectra were first pre-processed using rubber band baseline correction (RBC) in Orange data mining V. 9.1, 64 Bit [51] over the spectral regions 3100–2650 cm^−1^ and 1800–660 cm^−1^. This baseline correction was selected due to the non-linear nature of the baseline observed in these data. This was followed by standard normal variate (SNV) transformation in The Unscrambler X 10.5.1 (CAMO, Oslo, Norway).

IR spectral data were imported into The Unscrambler X 10.5.1 for pre-processing and analysis. IR spectra were pre-processed using SNV over the spectral windows 3050–2600 cm^−1^ and 1800 to 300 cm^−1^. These regions were selected to avoid the diamond window signals and because they contained the greatest amount of meaningful data about the samples [52].

Low-level data fusion was carried out using the pre-processed spectral data from the two different instruments (Raman and IR spectroscopy) and creating a single matrix by concatenating the two spectral datasets [30,38,53,54].

Qualitative, classification and quantitative analysis were performed to explore the variation in the dataset, identify and classify the adulterated samples and quantify the level of adulteration. All multivariate analyses described here were performed using The Unscrambler X 10.5.1 (CAMO, Oslo, Norway).

Unsupervised evaluation of the dataset was carried out using principal component analysis (PCA) to explore the variation described in the datasets. PCA was carried out on the pre-processed datasets using the Raman spectral ranges 3100–2650 cm^−1^ and 1800–660 cm^−1^ and IR spectral ranges 3050–2600 cm^−1^ and 1800–300 cm^−1^ with the non-linear iterative partial least squares (NIPALS) algorithm and full “leave-one-out” cross-validation.

Support vector machine (SVM) classification was carried out to identify and classify the presence and type of adulterant in the krill oil samples. Based on this classification, the appropriate adulterant-specific quantitative model can be applied to the unknown sample. The spectra were divided into model and test sets based on the experimental design described above in Section 2.1. The SVM classification models were developed using the model set data with four classes (unadulterated, adulterated with PO, adulterated with O3C and adulterated with FO). The independent test set data were used to evaluate the SVM classification model performance. The SVM classification models were developed for both an individual (either Raman or IR spectroscopic data) and a data fused approach. The SVM classification models were calculated using a C-SVC type SVM with a radial basis function (RBF) for the IR spectroscopic data or a linear kernel function for the Raman and fused data with a gamma coefficient of 0.1 and a C-value of 100, using The Unscrambler X 10.5.1 (CAMO, Oslo, Norway). The SVM models were evaluated using the independent test set data to obtain classification accuracies as a measure for the model performance.

Partial least square regression (PLSR) models were developed to quantify the amount of adulterant present in oil mixtures over the same spectral range as described earlier [30]. For PLSR model development, the pre-processed spectral data (*X*-matrix) were correlated against the concentration values (% by weight) of the adulterant present in the oil mixtures (PO, O3C and FO) as reference data (*Y*-matrix) using a NIPALS algorithm and full “leave-one-out” cross-validation. The PLSR model performances were evaluated using the root mean squared error of prediction (RMSEP) value from the independent test set data.

## 4. Conclusions

The present study investigated the feasibility of using Raman and IR spectroscopy in combination with chemometrics to detect and quantify the adulteration of cheaper oils (PO, O3C and FO) in krill oil samples. IR spectroscopy performed better than Raman spectroscopy for the quantificati on of PO (3.5% RMSEP) and FO (9.6% RMSEP) adulteration in krill oil. Raman provided better quantitative prediction of O3C adulteration in krill oil (2.3% RMSEP) compared to IR spectroscopy. However, low-level data fusion of the two techniques provided further model improvement for the quantification of PO (2.7% RMSEP) and FO (9.1% RMSEP) in KO. This study therefore demonstrates the potential of using of Raman and IR spectroscopic techniques for the non-destructive detection and quantification of adulteration of krill oil samples.

## Figures and Tables

**Table 1:** The model performance of PLSR for quantification of PO, O3C and FO oils as oil adulterants in krill oil. The *italicized* rows indicate the best-performing model for each oil type.

Adulterant	Method	No. Factors	Calibration		Prediction (Test Set)				Prediction (Test Set) SVM			
			r^2^	RMSEC (%)	r^2^	Slope	Offset	RMSEP (%)	r^2^	Slope	Offset	RMSEP (%)
PO%	R	3	0.89	5.6	0.86	0.76	10.7	7.0	0.85	0.77	10.3	7.7
	I	3	0.97	2.4	0.96	3.7	0.97	3.5	0.96	1.0	0.31	3.5
	F	2	0.94	4.2	0.97	0.93	3.3	3.6	*0.98*	*0.96*	*1.9*	*2.7*
O3C%	R	4	0.98	2.4	0.98	1.1	−1.5	2.4	*0.95*	*0.96*	*2.8*	*2.3*
	I	2	0.91	5.3	0.96	0.87	1.2	3.5	0.95	0.83	2.7	4.2
	F	3	0.96	3.7	0.96	1.0	1.2	3.3	0.97	0.94	2.8	3.0
FO%	R	4	0.95	3.4	0.90	0.92	6.3	5.8	0.31	0.59	18.8	15.6
	I	3	0.90	5.0	0.78	0.85	−3.1	8.4	0.73	0.72	0.12	9.6
	F	2	0.82	6.7	0.75	0.84	−3.1	9.1	*0.76*	*0.89*	*−5.2*	*9.1*

Methods R = Raman, I = infrared, F = fused. RMSEC = Root means square error of calibration, and RMSEP = Root means square error of prediction.

## Data Availability

Data are available from the authors.

## Supplementary Materials

The following supporting information can be downloaded at: https://www.mdpi.com/article/10.3390/molecules28093695/s1, Figure S1: An overview of all oil samples used in this experiment. Abbreviation: KO = krill oil; PO = palm oil; O3C = ω-3 concentrates in ethyl ester; FO = fish oil; Figure S2: Decision tree/workflow for evaluating unknown samples; Figure S3: PLSR calibration lines and regression coefficients for quantitative prediction of FO concentration in krill oil by Raman (a,b), IR (c,d) and low-level fused Raman plus IR data (e,f); Table S1: A summary of the studied samples with sample name, country of origin, company, batch number and best before; Table S2: Summary of the sample mixtures used in this study. Samples are expressed as weight percentages (% *w*/*w*). Abbreviations: VO = valuable oil; KO1 to KO6 = Krill oil batch; PO = palm oil; O3C = ω-3 concentrates in ethyl ester; FO = Fish oil; M = model set; T = test set; Table S3: SVM Model performance for classification of pure (KO) and adulterants (PO, O3C and FO) using FT-Raman, FT-IR and Fused data; Table S4: SVM model performance with accuracy, sensitivity and specificity test of pure (KO) and adulterants (PO, O3C and FO) using FT-Raman, FT-IR and Fused data.

## Sample Availability

Samples are no longer available as they have been discarded.
