# Supplementary material for: Detection and Quantification of Adulteration in Krill Oil with Raman and Infrared Spectroscopic Methods
Source: Molecules. 2023 Apr 25;28(9):3695. doi: 10.3390/molecules28093695 (PMC10180486; doi:10.3390/molecules28093695)
Supplement: Supplementary file 1 [file molecules-28-03695-s001.zip › molecules-2339788-supplementary.pdf]

Supplementary information for:

Detection and Quantification of Adulteration in Krill Oil with Raman and Infrared Spectroscopic Methods

Fatema Ahmmed [1,†], Keith C. Gordon [1,*], Daniel P. Killeen [2] and Sara J. Fraser-Miller [1,*]

1 Te Whai Ao-Dodd-Walls Centre for Photonic and Quantum Technologies, Department of Chemistry, University of Otago, P.O. Box 56, Dunedin 9016, New Zealand; fahmmed@massey.ac.nz
2 The New Zealand Institute for Plant and Food Research Limited, P.O. Box 5114, Port Nelson, Nelson 7043, New Zealand; daniel.killeen@plantandfood.co.nz
* Correspondence: keith.gordon@otago.ac.nz (K.C.G.); sara.miller@otago.ac.nz (S.J.F.-M.)
† Current address: Riddet Institute, Massey University, Private Bag 11 222, Palmerston North 4442, New Zealand.

List of Contents

Figure S1 : An overview of all oil samples used in this experiment. Abbreviation: KO = krill oil; PO = palm oil; O3C = ω-3 concentrates in ethyl ester; FO = fish oil.......... 2

Figure S2 : Decision tree/workflow for evaluating unknown samples.......... 3

Figure S3 : PLSR calibration lines and regression coefficients for quantitative prediction of FO concentration in krill oil by Raman (a,b), IR (c,d) and low-level fused Raman plus IR data (e,f).......... 4

Table S1: A summary of the studied samples with sample name, country of origin, company, batch number and best before.......... 5

Table S2: Summary of the sample mixtures used in this study. Samples are expressed as weight percentages (% w/w). Abbreviations: VO = valuable oil; KO1 to KO6 = Krill oil batch; PO = palm oil; O3C = ω-3 concentrates in ethyl ester; FO = Fish oil; M = model set; T = test set.......... 6

Table S3: SVM Model performance for classification of pure (KO) and adulterants (PO, O3C and FO) using FT-Raman, FT-IR and Fused data.......... 9

Table S4: SVM model performance with accuracy, sensitivity and specificity test of pure (KO) and adulterants (PO, O3C and FO) using FT-Raman, FT-IR and Fused data.......... 10

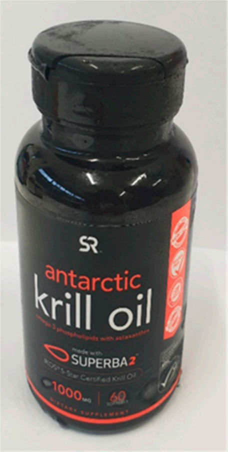

K1

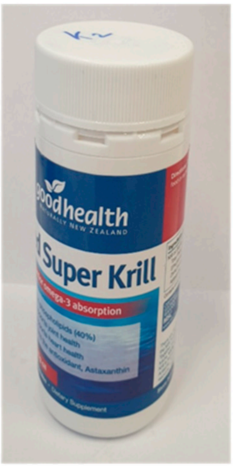

K2

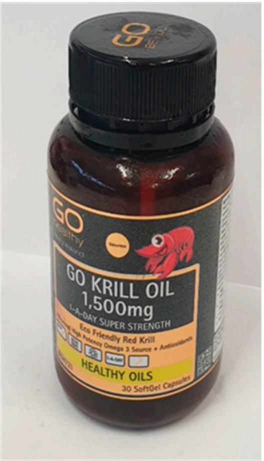

K3

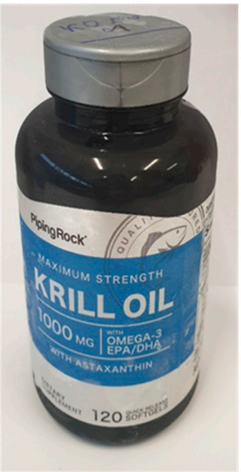

K4

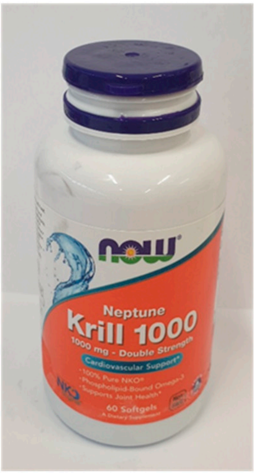

K5

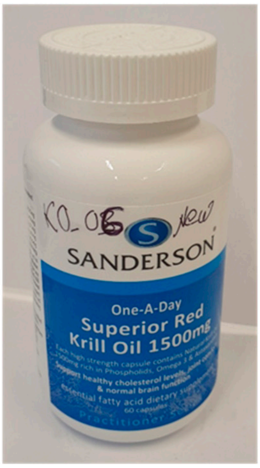

K6

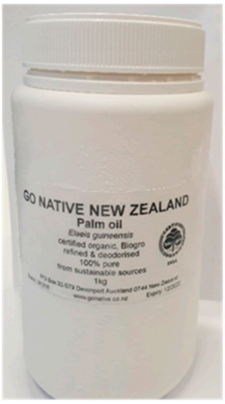

PO

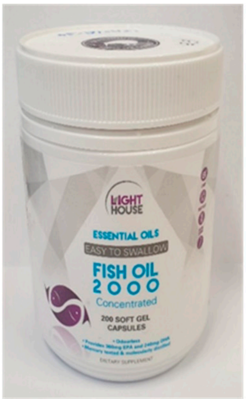

O3C

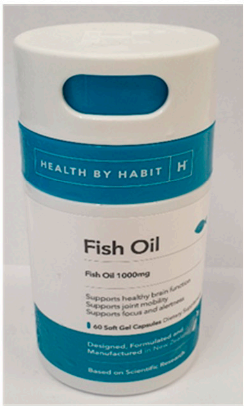

FO

Figure S1 : An overview of all oil samples used in this experiment. Abbreviation: KO = krill oil; PO = palm oil; O3C = ω-3 concentrates in ethyl ester; FO = fish oil

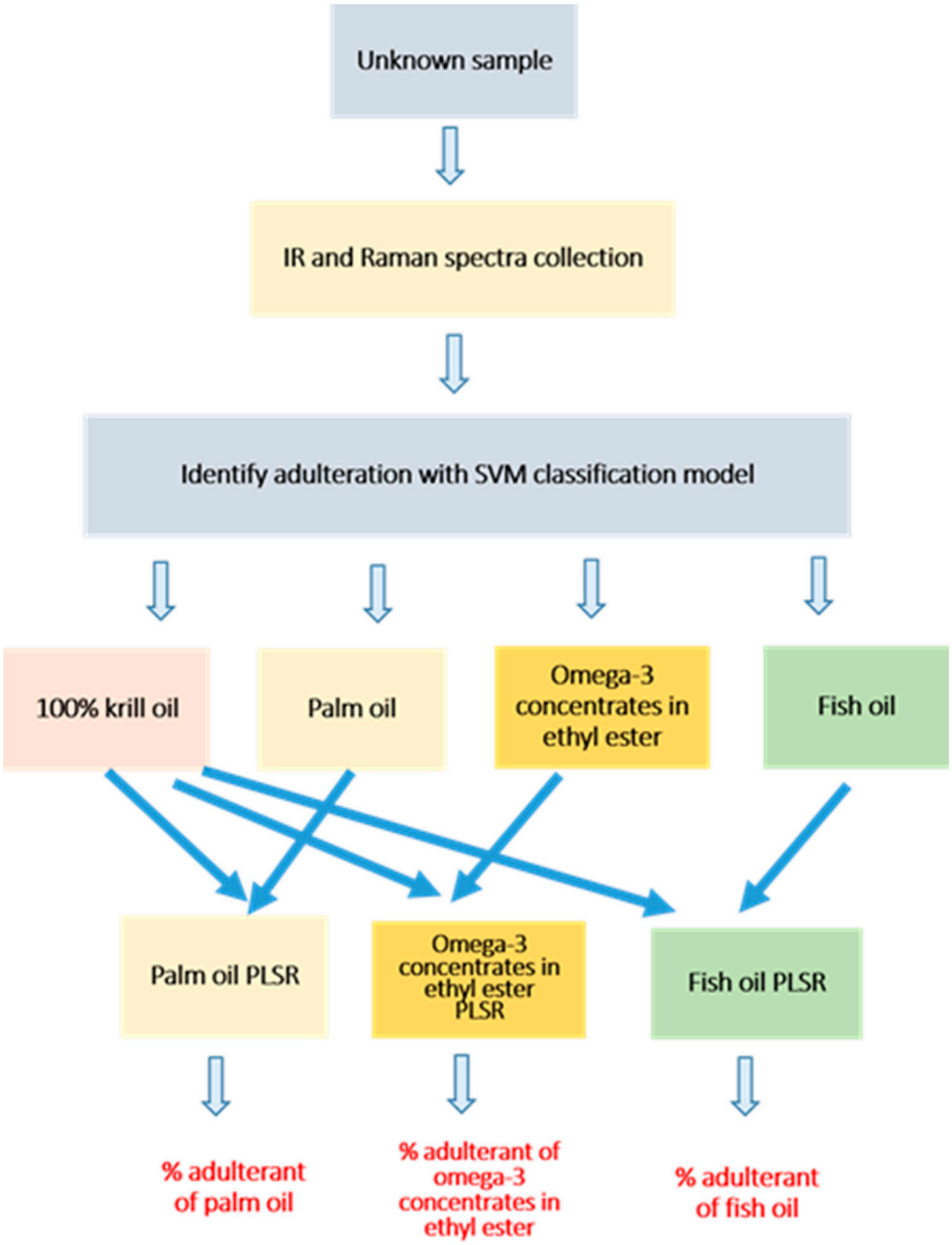

Figure S2 : Decision tree/workflow for evaluating unknown samples

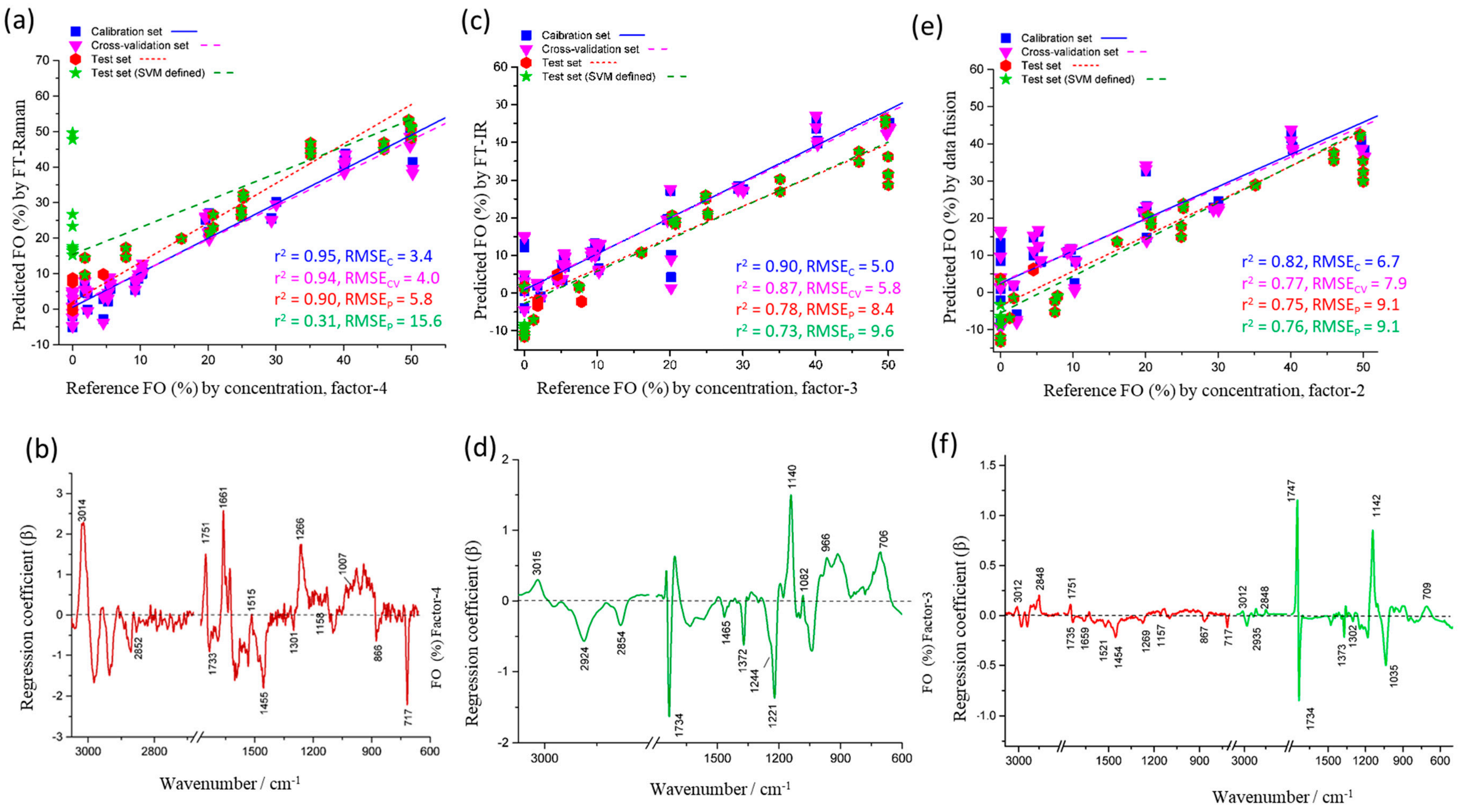

Figure S3 : PLSR calibration lines and regression coefficients for quantitative prediction of FO concentration in krill oil by Raman (a,b), IR (c,d) and low-level fused Raman plus IR data (e,f)

Table S1: A summary of the studied samples with sample name, country of origin, company, batch number and best before

Sample name	Country of origin	company	batch no.	best before
**Antarctic krill oil (KO1)**	New Zealand	SR	SRE-00453	07/2023
**Red super krill (KO2)**	New Zealand	Good health	P90L	03/2023
**Go Krill oil (KO3)**	New Zealand	Go healthy	JO270	01/2023
**Krill oil (KO4)**	New Zealand	PipingRock	B980E	08/2023
**Neptune krill 1000 (KO5)**	USA	NOW	1627BT	06/2023
**Superior red krill oil (KO6)**	New Zealand	Sanderson	B7629	5/2024
**Fish oil 2000 concentrated (O3C)**	New Zealand	Lighthouse	T8005	09/2021
**Palm oil (PO)**	New Zealand	Go native New Zealand	01216	12/2022
**Fish oil (FO)**	New Zealand	Health by habit	KO875	07/2023

Table S2: Summary of the sample mixtures used in this study. Samples are expressed as weight percentages (% w/w). Abbreviations: VO = valuable oil; KO1 to KO6 = Krill oil batch; PO = palm oil; O3C = ω-3 concentrates in ethyl ester; FO = Fish oil; M = model set; T = test set

Predominant oil type	Batch	VO (% w/w)	PO (% w/w)	O3C (% w/w)	FO (% w/w)	Dataset (M or T)
Krill oil samples	KO1	100.0	0.0	0.0	0.0	M
	KO1	97.8	0.0	0.0	2.2	M
	KO1	89.8	0.0	0.0	10.2	M
	KO1	70.0	0.0	0.0	30.0	M
	KO1	50.2	0.0	0.0	49.8	M
	KO1	94.9	0.0	5.1	0.0	M
	KO1	79.6	0.0	20.4	0.0	M
	KO1	97.8	2.2	0.0	0.0	M
	KO1	89.2	10.8	0.0	0.0	M
	KO1	69.6	30.4	0.0	0.0	M
	KO1	51.6	48.4	0.0	0.0	M
	KO1	60.0	0.0	40.0	0.0	M
	KO1	79.9	0.0	20.1	0.0	M
	KO2	100.0	0.0	0.0	0.0	M
	KO2	94.5	0.0	0.0	5.5	M
	KO2	94.4	0.0	0.0	5.6	M
	KO2	90.8	0.0	0.0	9.2	M
	KO2	90.3	0.0	0.0	9.7	M
	KO2	80.4	0.0	0.0	19.6	M
	KO2	59.7	0.0	0.0	40.3	M
	KO2	97.4	0.0	2.6	0.0	M
	KO2	89.8	0.0	10.2	0.0	M
	KO2	70.6	0.0	29.4	0.0	M
	KO2	50.1	0.0	49.9	0.0	M
	KO2	50.0	0.0	50.0	0.0	M
	KO2	94.7	5.3	0.0	0.0	M
	KO2	79.6	20.4	0.0	0.0	M
	KO2	75.8	24.2	0.0	0.0	M
	KO2	75.0	25.0	0.0	0.0	M
	KO2	60.7	39.3	0.0	0.0	M
	KO2	59.9	40.1	0.0	0.0	M
	KO4	100.0	0.0	0.0	0.0	M

	KO4	98.2	0.0	0.0	1.8	M
	KO4	89.6	0.0	0.0	10.4	M
	KO4	70.7	0.0	0.0	29.4	M
	KO4	49.8	0.0	0.0	50.2	M
	KO4	93.6	0.0	6.4	0.0	M
	KO4	80.1	0.0	19.9	0.0	M
	KO4	60.6	0.0	39.4	0.0	M
	KO4	98.0	2.0	0.0	0.0	M
	KO4	90.3	9.7	0.0	0.0	M
	KO4	70.4	29.6	0.0	0.0	M
	KO4	49.7	50.3	0.0	0.0	M
	KO6	100.0	0.0	0.0	0.0	M
	KO6	95.5	0.0	0.0	4.5	M
	KO6	79.9	0.0	0.0	20.1	M
	KO6	59.9	0.0	0.0	40.1	M
	KO6	98.0	0.0	2.0	0.0	M
	KO6	89.9	0.0	10.1	0.0	M
	KO6	70.9	0.0	29.1	0.0	M
	KO6	50.3	0.0	49.7	0.0	M
	KO6	94.4	5.6	0.0	0.0	M
	KO6	79.9	20.1	0.0	0.0	M
	KO6	60.1	39.9	0.0	0.0	M
	KO6	80.2	19.8	0.0	0.0	M
	KO6	79.8	0.0	0.0	20.2	M
	KO6	90.3	0.0	9.7	0.0	M
	KO6	94.7	5.3	0.0	0.0	M
	KO6	94.8	0.0	0.0	5.2	M
	KO6	99.1	0.0	0.9	0.0	M
	KO3	100.0	0.0	0.0	0.0	T
	KO3	95.5	0.0	0.0	4.5	T
	KO3	83.9	0.0	0.0	16.1	T
	KO3	79.7	0.0	0.0	20.3	T
	KO3	79.2	0.0	0.0	20.8	T
	KO3	74.8	0.0	0.0	25.2	T
	KO3	54.1	0.0	0.0	45.9	T
	KO3	50.0	0.0	0.0	50.0	T
	KO3	95.6	0.0	4.4	0.0	T
	KO3	90.8	0.0	9.2	0.0	T
	KO3	74.5	0.0	25.5	0.0	T
	KO3	70.1	0.0	29.9	0.0	T
	KO3	69.8	0.0	30.2	0.0	T

	KO3	64.7	0.0	35.3	0.0	T
	KO3	50.2	0.0	49.8	0.0	T
	KO3	84.5	15.5	0.0	0.0	T
	KO3	74.9	25.1	0.0	0.0	T
	KO3	54.1	45.9	0.0	0.0	T
	KO3	49.5	50.5	0.0	0.0	T
	KO3	50.0	0.0	0.0	50.0	T
	KO5	100.0	0.0	0.0	0.0	T
	KO5	98.2	0.0	0.0	1.8	T
	KO5	92.1	0.0	0.0	7.9	T
	KO5	75.1	0.0	0.0	24.9	T
	KO5	64.9	0.0	0.0	35.1	T
	KO5	50.4	0.0	0.0	49.6	T
	KO5	98.3	0.0	1.7	0.0	T
	KO5	85.0	0.0	15.0	0.0	T
	KO5	75.6	0.0	24.4	0.0	T
	KO5	55.3	0.0	44.7	0.0	T
	KO5	49.7	0.0	50.3	0.0	T
	KO5	98.5	1.5	0.0	0.0	T
	KO5	92.4	7.6	0.0	0.0	T
	KO5	75.2	24.9	0.0	0.0	T
	KO5	65.3	34.7	0.0	0.0	T
	KO5	49.8	50.2	0.0	0.0	T
	KO5	92.5	0.0	0.0	7.5	T
	KO5	98.8	0.0	0.0	1.2	T

Table S3: SVM Model performance for classification of pure (KO) and adulterants (PO, O3C and FO) using FT-Raman, FT-IR and Fused data

	**FT-Raman**	**Test set (76% accuracy)**			
**Confusion matrix**	Actual	1	2	3	4
**Predicted**		KO	PO	O3C	FO
**1**	KO	0	0	0	2
**2**	PO	4	18	0	4
**3**	O3C	0	0	17	0
**4**	FO	0	0	7	19
	**FT-IR**	**Test set (79% accuracy)**			
**Confusion matrix**	Actual	1	2	3	4
**Predicted**		KO	PO	O3C	FO
**1**	KO	2	4	2	4
**2**	PO	2	13	0	2
**3**	O3C	0	0	22	1
**4**	FO	0	0	0	18
	**Fused data**	**Test set (84% accuracy)**			
**Confusion matrix**	Actual	1	2	3	4
**Predicted**		KO	PO	O3C	FO
**1**	KO	4	2	3	4
**2**	PO	0	16	0	1
**3**	O3C	0	0	20	0
**4**	FO	0	0	1	19

Table S4: SVM model performance with accuracy, sensitivity and specificity test of pure (KO) and adulterants (PO, O3C and FO) using FT-Raman, FT-IR and Fused data

Technique	Training set		Test set								
	Accuracy (calibration)	Accuracy (cross validation)	Accuracy	Sensitivity to individual classes (%)				Specificity to individual classes (%)			
				KO	PO	O3C	FO	KO	PO	O3C	FO
**FT-Raman**	100	86	76	0	100	71	76	97	85	100	85
**FT-IR**	100	96	79	50	76	92	72	85	92	98	100
**Fused data**	100	92	84	100	89	83	79	86	98	100	98
